# SONOlysis in prevention of Brain InfaRctions During Internal carotid Endarterectomy (SONOBIRDIE) trial – study protocol for a randomized controlled trial

**DOI:** 10.1186/s13063-016-1754-x

**Published:** 2017-01-17

**Authors:** Tomáš Hrbáč, David Netuka, Vladimír Beneš, Vladimír Nosáľ, Petra Kešnerová, Aleš Tomek, Táňa Fadrná, Vladimír Beneš, Jiří Fiedler, Vladimír Přibáň, Miroslav Brozman, Kateřina Langová, Roman Herzig, David Školoudík

**Affiliations:** 1 Department of Neurosurgery, Comprehensive Stroke Center, University Hospital Ostrava, Ostrava, Czech Republic; 2 Department of Neurosurgery and Neurooncology, Comprehensive Stroke Center, Military University Hospital, Prague, Czech Republic; 3 Department of Neurology, Jessenius Faculty of Medicine in Martin, Comenius University, Bratislava, Slovak Republic; 4 Department of Neurology, Comprehensive Stroke Center, 2nd Faculty of Medicine, Charles University in Prague and Motol University Hospital, Prague, Czech Republic; 5 Center for Research and Science, Faculty of Health Sciences, Palacký University Olomouc, Olomouc, Czech Republic; 6 Department of Neurosurgery, Comprehensive Stroke Center, Liberec Hospital, Liberec, Czech Republic; 7 Department of Neurosurgery, Comprehensive Stroke Center, České Budějovice Hospital, České Budějovice, Czech Republic; 8 Department of Neurosurgery, Comprehensive Stroke Center, University Hospital Plzeň, Plzeň, Czech Republic; 9 Department of Neurology, Faculty Hospital Nitra, Constantine Philosopher University Nitra, Nitra, Slovakia; 10 Department of Biophysics, Faculty of Medicine and Dentistry, Institute of Molecular and Translational Medicine, Palacký University, Olomouc, Czech Republic; 11 Department of Neurology, Comprehensive Stroke Center, Charles University Faculty of Medicine and University Hospital Hradec Králové, Hradec Králové, Czech Republic; 12 Department of Neurology, Comprehensive Stroke Center, University Hospital Ostrava, Ostrava, Czech Republic

**Keywords:** Sonolysis, Stroke, Carotid, Endarterectomy, Brain infarction

## Abstract

**Background:**

Carotid endarterectomy (CEA) is a beneficial procedure for selected patients with an internal carotid artery (ICA) stenosis. Surgical risk of CEA varies from between 2 and 15%.

The aim of the study is to demonstrate the safety and effectiveness of sonolysis (continual transcranial Doppler monitoring, TCD) using a 2-MHz diagnostic probe with maximal diagnostic energy on the reduction of the incidence of stroke, transient ischemic attack (TIA) and brain infarction detected using magnetic resonance imaging (MRI) by the activation of the endogenous fibrinolytic system during CEA.

**Methods/design:**

Design: a multicenter, randomized, double-blind, sham-controlled trial.

Scope: international, multicenter trial for patients with at least 70% symptomatic or asymptomatic ICA stenosis undergoing CEA.

Inclusion criteria: patients with symptomatic or asymptomatic ICA stenosis of at least 70% are candidates for CEA; a sufficient temporal bone window for TCD; aged 40–85 years, functionally independent; provision of signed informed consent.

Randomization: consecutive patients will be assigned to the sonolysis or control (sham procedure) group by computer-generated 1:1 randomization. Prestudy calculations showed that a minimum of 704 patients in each group is needed to reach a significant difference with an alpha value of 0.05 (two-tailed) and a beta value of 0.8 assuming that 10% would be lost to follow-up or refuse to participate in the study (estimated 39 endpoints).

Endpoints: the primary endpoint is the incidence of stroke or TIA during 30 days after CEA and the incidence of new ischemic lesions on brain MRI performed 24 h after CEA in the sonolysis and control groups. Secondary endpoints are occurrence of death, any stroke, or myocardial infarction within 30 days, changes in cognitive functions 1 year post procedure related to pretreatment scores, and number of new lesions and occurrence of new lesions ≥0.5 mL on post-procedural brain MRI.

Analysis: descriptive statistics and linear/logistic multiple regression models will be performed. Clinical relevance will be measured as relative risk reduction, absolute risk reduction and the number needed to treat.

**Discussion:**

Reduction of the periprocedural complications of CEA using sonolysis as a widely available and cheap method may significantly increase the safety of CEA and extend the indication criteria for CEA.

**Trial registration:**

ClinicalTrials.gov, NCT02398734. Registered on 20 March 2015.

**Electronic supplementary material:**

The online version of this article (doi:10.1186/s13063-016-1754-x) contains supplementary material, which is available to authorized users.

## Background

Stroke is the third most common cause of death in the majority of the developed countries [1]. This has also been so in the Czech Republic for many years – approximately 7000 men and 10,000 women die of stroke each year [2]. Stroke in the Czech Republic has even double the incidence than the remainder of Western Europe. Internal carotid artery (ICA) stenosis is one of the most common etiological factors of ischemic stroke, causing 10–35% of strokes [3].

Stroke risk increases with the increased severity of ICA stenosis and this risk is higher in symptomatic stenoses than in asymptomatic ones. Results of the NASCET [4], ECST [5] and ACAS [6] studies have shown that carotid endarterectomy (CEA) represents a beneficial procedure for patients with a symptomatic ICA stenosis of >50% and for patients with an asymptomatic ICA stenosis >60% [7]. Perioperative risk of transient ischemic attack (TIA), stroke, myocardial infarction or death for CEA varies between 2 and 15% [3, 8]. However, even clinically silent microembolism can cause microinfarctions presenting with postoperative cognitive deficit [9]. Carotid artery stenting represents another treatment possibility in these patients and patients with contraindications to CEA or high-risk patients are the main candidates for carotid artery stenting [7, 10]. Nevertheless, CEA remains the “gold standard” for ICA stenosis treatment [11].

Since the 1970s, in-vitro and animal model studies have demonstrated acceleration of thrombus dissolution using an ultrasound beam. Studies with many animal models (e.g., rabbits, rats) showed acceleration of spontaneous or pharmacologically induced thrombolysis using ultrasound beam with frequencies ranging between 20 kHz and 2 MHz [12–14].

Alexandrov et al. cited a large number of early arterial recanalizations in acute stroke patients with middle cerebral artery (MCA) occlusion who were treated using systemic thrombolysis in combination with transcranial Doppler (TCD) monitoring [15].

Between 2002 and 2005, three other studies demonstrated the potential effect of diagnostic ultrasound on the acceleration of spontaneous or induced recanalization of intracranial arteries [16–18]. This therapeutic procedure is called sonolysis or thrombotripsy when ultrasound monitoring is used alone or sonothrombolysis when ultrasound monitoring is used in combination with thrombolytics. There are two possible effects of ultrasound on thrombus: (1) mechanical destruction due to vibration of the thrombus with acceleration of penetration of fibrinolytics into the thrombus and (2) elevation of temperature and stimulation of endothelium with local activation of the fibrinolytic system [16–21]. A recent study showed that TCD monitoring had a significant effect on activation of the fibrinolytic system in healthy volunteers [22].

TCD monitoring during CEA is a common diagnostic method being used for the detection of microemboli and changes of blood flow in intracranial stenoses [23]. Reduction of periprocedural complications of CEA with TCD monitoring was mentioned in some studies. This reduction of stroke risk could be due to the sophisticated indications for shunt implementation, optimization of surgery and anesthesia according to the blood flow changes, and the detection of microemboli in the MCA using TCD monitoring [23]. Another cause of the reduction of microinfarctions is the local activation of the endogenous fibrinolytic system due to TCD monitoring (equal to sonothrombolysis in acute stroke studies) [21]. The recently published SONOBUSTER trial showed that intraoperative sonolysis reduced both the incidence and the volume of new brain infarctions following CEA. These benefits were most evident for larger infarctions (volume ≥0.5 mL) and extended beyond the region directly exposed to ultrasonic waves (i.e., the contralateral hemisphere) [22]. However, the effect on cognitive function decline was not significant in this study due to the low number of patients involved [22].

## Methods/design

### Study objectives

The objective of this multicenter, randomized, double-blind, sham-controlled study is to demonstrate the safety and effectiveness of sonolysis (continual TCD monitoring), using a 2-MHz diagnostic probe with a maximal diagnostic energy, on the reduction of the incidence of stroke, TIA and brain infarction by activation of the endogenous fibrinolytic system during CEA in patients with ≥70% symptomatic or asymptomatic stenosis of the ICA.

The substudy aims to compare the risk of brain infarction detected using magnetic resonance imaging (MRI) between the sonolysis and the control group.

### Overview

The SONOBIRDIE trial is a randomized, double-blind, sham-controlled study designed to demonstrate the safety and effectiveness of sonolysis (continual TCD monitoring) in reduction of the risk of stroke or TIA, brain infarction and cognitive decline using a 2-MHz diagnostic probe with a maximal diagnostic energy on the reduction of the risk of brain infarction by the activation of endogenous fibrinolytic system during CEA in patients with ≥70% symptomatic or asymptomatic ICA stenosis (Table 1).

**Table 1:** SONOBIRDIE trial: the schedule of enrollment, interventions and assessments

	Visit 1 Baseline and randomization	Visit 2 (maximum 24 h before CEA)	Allocation (during CEA)	Visit 3 (24–48 h after CEA)	Visit 4 (30 ± 2 days after CEA)	Visit 5 (365 ± 14 days after CEA)
Medical and surgical history	X	X		X	X	X
Demographics	X					
Physical examination	X	X		X	X	X
Vital signs	X	X				X
Neurological examination	X	X		X	X	X
NIHSS	X	X		X	X	X
mRS	X	X		X	X	X
Duplex sonography	X	X		X	X	X
CTA, MRA or DSA	X					
Signed informed consent	X					
Eligibility criteria	X					
MRI (for substudy only)		X		X (24 ± 4 h)		
ACE-R		X		X	X	X
MMSE		X		X	X	X
Clock-drawing Test		X		X	X	X
Verbal Fluency Test		X		X	X	X
Sonolysis			X			
Sham procedure			X			
Adverse event, SAE				X	X	X
Endpoints				X	X	X

*ACE-R* Addenbrooke’s Cognitive Examination Revised, *CEA* carotid endarterectomy, *CTA* computed tomography angiography, *DSA* digital subtraction angiography, *MMSE* Mini Mental State Examination, *MRA* magnetic resonance angiography, *MRI* magnetic resonance imaging, *mRS* modified Rankin score, *NIHSS* National Institutes of Health Stroke Scale, *SAE* serious adverse event

### Ethical approval of the study protocol

The study is conducted in accordance with the Declaration of Helsinki of 1975 (as revised in 2004 and 2008) and approved by the multicenter Ethics Committee of Vítkovice Hospital and local Ethics Committees of all participating centers. All patients provide written informed consent before enrollment. The study design was registered before the first patient enrollment at https://www.clinicaltrials.gov/ct2/show/NCT02398734?term=SONOBIRDIE&rank=1 (NCT02398734).

### Expected sample size

The sample size is based on an expected 2.5% absolute risk reduction of stroke or TIA incidence during the 30-day postoperative period in the sonolysis group (estimated prevalence, 1.5%) compared to the control group (estimated prevalence, 4%). Prestudy calculations showed that a minimum of 704 patients in each group is needed to reach a significant difference with an alpha value of 0.05 (two-tailed) and a beta value of 0.8 assuming that 10% would be lost to follow-up or refuse to participate in the study (estimated 39 endpoints).

Due to the higher sensitivity of MRI in the detection of new brain ischemic lesions, the sample size for the MRI substudy was based on an expected 15% reduction of new ischemic lesions on diffusion-weighted imaging-magnetic resonance imaging (DWI-MRI) in the sonolysis group (estimated prevalence, 10%) compared to the control group (estimated prevalence, 25%). Prestudy calculations showed that a minimum of 124 patients undergoing brain MRI in each group was needed to reach a significant difference with an alpha value of 0.05 (two-tailed) and a beta value of 0.8 assuming that 10% would be lost to follow-up or refuse to participate in the MRI substudy.

### Inclusion criteria

1. Subject has a symptomatic or asymptomatic ICA stenosis ≥70% using NASCET criteria as detected by a duplex sonography and confirmed using computed tomography angiography (CTA), magnetic resonance angiography (MRA) or digital subtraction angiography (DSA) [4]
2. Subject has indications for CEA according to the criteria set by the American Heart Association/American Stroke Association and Czech guidelines [7, 24]
3. Subject is aged 40–85 years
4. Subject has a sufficient temporal bone window for TCD with detectable blood flow in the MCA
5. Subject is functionally independent with a modified Rankin score (mRS) value of 0–2 points
6. Informed consent is signed by the subject

### Exclusion criteria

1. Subject has been participating in another clinical trial within last 6 weeks
2. Subject has any other medical condition that would make them inappropriate for study participation in the opinion of the investigator

### Substudy

All patients who fulfill the main study criteria and will have no contraindication to MRI (e.g., pacemaker in situ, implanted metal material, claustrophobia) will be enrolled in the MRI substudy until 124 patients have been assigned to each group.

### Test device

The TCD systems (e.g., DWL Multi-Dop T1, DWL, Sipplingen, Germany) with a diagnostic 2-MHz probe will be used for sonolysis (nondiagnostic TCD monitoring).

### Sonolysis

In patients randomized into the sonolysis group, a MCA segment to a depth of 55 mm will be continuously monitored during intervention using a diagnostic 2-MHz TCD probe with a maximal diagnostic energy (Thermal Index for cranial bone (TIC) approximately 1.3) and a sample volume of 10 mm. The probe will be fixed in the required position using a special helmet and sonolysis will start before the carotid intervention and will be stopped after the intervention, but at latest after 120 min. A TCD machine (DWL MultiDop T1, DWL Elektronische Systeme Sipplingen, Germany) with a 2-MHz diagnostic TCD probe will be used. This nondiagnostic TCD monitoring will be performed with recording of microembolic signals and changes in blood flow. Device sound and Doppler wave imaging will be switched off. Only the sonographer will be unblinded to the procedure.

### Sham procedure

In patients randomized into the control group, the TCD probe will be fixed in the required position using a special helmet as in sonolysis group patients, but a MCA segment to a depth of 55 ± 5 mm will be localized using a diagnostic 2-MHz transcranial TCD probe with a maximal diagnostic energy and the TCD monitoring will be stopped afterwards. Patients in the control group will undergo a sham procedure in which further sonolysis (TCD monitoring) will not be conducted.

### Carotid endarterectomy

Surgery will be performed under general or local anesthesia (the decision will be left to the discretion of the operating team) using an incision in front of the angle of the sternomastoid muscle. The common carotid artery and later the ICA and the external carotid artery will be mobilized. The common carotid artery, the ICA and the external carotid artery will be temporarily closed. Using a longitudinal incision of the common carotid artery and the ICA, atherosclerotic plaque will be visualized. Plaque will be withdrawn under microscopic control and later an arteriotomy suture will be performed using a monofilament nonabsorbent 6/0 fiber. Just before the end of surgery, hemostasis will be achieved and a drainage mechanism applied. Surgery will be completed by suture of subcutis and cutis. Unfractionated heparin (100 IU/kg bodyweight) may be administered to all patients just before the arteriotomy. In the case of insufficient collateral flow into the MCA after clipping of the common carotid artery and the ICA, a temporal shunt may be used. Antiplatelet therapy (clopidogrel 75 mg/day or acetylsalicylic acid 100 mg/day) will be used continuously in all patients. The surgeon will be blinded to the sonolysis or sham procedures.

### Magnetic resonance imaging

Magnetic resonance imaging (MRI) will be performed in patients enrolled in the MRI substudy. The MRI protocol consists of four sequences: (1) transverse T2-weighted spin echo (echo time, 100 ms; repetition time, 4310 ms; section thickness, 5.0 mm; matrix size, 192 × 256; gap, 0.5 mm; field of view (FOV), 250 mm; FOV ph, 75%; echo train length (ETL), 9; number of excitations, 1); (2) diffusion-weighted imaging (echo time, 130 ms; repetition time, 4500 ms; *b*, representing a factor of diffusion-weighted sequences *b* = 0 and *b* = 1,000 s/mm^2^; section thickness, 5.0 mm; gap, 1 mm; matrix size, 192 × 192; FOV, 255 mm; FOV ph, 100%; number of excitations, 4; echo spacing, 0.93 ms; bandwidth, 1240 Hz/Px); apparent diffusion coefficient maps will be obtained in all cases; (3) T2 star-weighted gradient-recalled echo (GRE) sequence for detection of bleeding (including microbleeds); (4) fluid-attenuated inversion recovery (FLAIR; echo time, 109 ms; repetition time, 8000 ms; inversion time, 2500 ms; section thickness, 5.0 mm; matrix size, 256 × 151; gap, 0.5 mm; FOV, 250 mm; FOV ph, 77%; number of excitations, 1; ETL, 5). Sequences will be applied at an identical level with the same slice thickness and identical cut number. Slice thickness will comprise the cut thickness (5 mm) + gap (10%). The standard number of slices will be 25. The standard slice level will be considered to be a modified level of the skull base due to minimalization of the distant-artifacts’ echo planar imaging (EPI) sequence.

In accordance with previous studies [22, 25–29], a new ischemic brain lesion is defined as hyperintense regions on the post-intervention DWI that were not present on pretreatment images. The volume of new brain infarctions will be measured manually. Infarct volumes will be calculated as the total hyperintense area in single slices multiplied by an effective slice thickness:

$$ \left[\left(\mathrm{Actual}\kern0.5em \mathrm{slice}\kern0.5em \mathrm{thickness}+\mathrm{Distance}\kern0.5em \mathrm{factor}\right)/\mathrm{Interslice}\kern0.5em \mathrm{gap}\right]. $$

Ischemic lesions in the brain will be evaluated by two blinded investigators. All disagreements will be resolved by consensus. The third blinded investigator will be involved in a case of a persistent disagreement. Ischemic lesions <0.5 mL or ≥0.5 mL will be evaluated separately in the subanalyses. New ischemic lesions in the brain will be classified as ipsilateral or contralateral to the treated vessel. Enlargement of a previous DWI lesion will be not considered a new ischemic lesion.

### Clinical examinations

Standard physical and neurological examinations will be performed before CEA, 24 h, 30 days and 1 year after CEA. Evaluation of the neurological deficit will be performed using the National Institutes of Health Stroke Scale (NIHSS) and mRS before CEA, 24 h, 30 days and 1 year after CEA.

### Cognitive tests

Cognitive testing (Addenbrooke’s Cognitive Examination Revised (ACE-R), the Mini Mental State Examination (MMSE), the Clock-drawing Test, the Verbal Fluency Test) will be performed before, 24 h, 30 days and 1 year after CEA.

The MMSE will be used to assess orientation, registration (immediate memory), short-term memory and language functioning. It includes 30 questions. One point will be given for each correct answer. The total results may range from 0 points as minimum to 30 points as maximum.

The Clock-drawing Test will be used for a brief cognitive task testing of executive functioning (memory, concentration, initiation, energy, mental clarity and indecision). Errors in clock-drawing will be evaluated: omissions, perseverations, rotations, misplacements, distortions, substitutions and additions. The Shulman scoring system (0–5 points) will be used for test result evaluation.

The Verbal Fluency Test will be used to test executive functioning and linguistic skills. Participants will be asked to say as many words beginning with the letter “P” as possible from a given category within 60 s. This will be scored from 0 to 7 points: 0–1 words, 0 points; 2–3 words, 1 point; 4–5 words, 2 points; 6–7 words, 3 points; 8–10 words, 4 points; 11–13 words, 5 points; 14–17 words, 6 points; and more than 17 words, 7 points.

All cognitive tests will be performed by a blinded investigator.

### Randomization

Consecutive patients will be assigned to the sonolysis or the control group by computer-generated 1:1 randomization.

### Data source

Datasets for analysis collected during visits will consist of:

A. Demographic data
  1. Age (years)
  2. Gender (male or female)
  3. Arterial hypertension (diagnosed)
  4. Diabetes mellitus (diagnosed)
  5. Coronary heart disease (diagnosed)
  6. Atrial fibrillation (diagnosed)
  7. Hyperlipidemia (diagnosed)
  8. Statin type
  9. Statin dose (milligrams per day)
  10. Smoking (number of cigarettes per day)
  11. Alcohol abuse (number of units per day)
  12. Antithrombotics (type)
  13. Antithrombotics (dose: milligrams per day)
B. Sonographic/computed tomography/angiography data
  1. Side of carotid stenosis
  2. Severity (%) of the intervened carotid stenosis – duplex sonography
  3. Severity (%) of the intervened carotid stenosis – CTA (NASCET)
  4. Severity (%) of the contralateral carotid stenosis – duplex sonography
  5. Severity (%) of the contralateral carotid stenosis – CTA (NASCET)
  6. Plaque characteristics
C. Intervention data
  1. Type of anesthesia (local, general)
  2. Time from symptom onset to intervention
  3. Sonolysis
  4. Duration of sonolysis
  5. Plaque extraction feasibility
  6. Shunt use
D. Clinical data (at visits 1, 2, 3, 4 and 5)
  1. NIHSS score
  2. mRS score
E. MRI data – endpoints (at visit 3)
  1. New brain infarction on DWI-MRI
  2. New brain infarction on DWI-MRI ipsilateral to the intervention
  3. New brain infarction with a total volume ≥0.5 cm^3^ (mL)
F. Cognitive tests data (at visits 2, 3, 4 and 5)
  1. MMSE result
  2. Clock-drawing Test result
  3. Verbal Fluency Test result
  4. ACE-R results
G. Clinical endpoints (at visits 3 and 4)
  1. Ischemic stroke or TIA within 30 days after the intervention
  2. Death within 30 days after the intervention
  3. Any stroke within 30 days after the intervention
  4. Myocardial infarction within 30 days after the intervention
H. Adverse events (at visits 3, 4 and 5)
  1. Adverse event during carotid intervention
  2. Adverse event during first 24 h after carotid intervention (e.g., worsening of neurological symptoms by ≥4 points in the NIHSS, brain edema, symptomatic and asymptomatic intracranial bleeding detected on control brain DWI-MRI)
  3. Adverse event during 30 days after carotid intervention (e.g., new admissions to the hospital, worsening of neurological symptoms by ≥4 points in the NIHSS)
I. Medication (at all visits)
  1. Antiplatelet therapy (generic name, daily dose)
  2. Anticoagulants (generic name, daily dose)
  3. Statin (generic name, daily dose)
  4. Antihypertensives (generic name, daily dose)
  5. Insulin
  6. Oral hypoglycemic agents
  7. Others

### Analysis sets

Efficacy analyses will be performed primary for the intent-to-treat population. The secondary analysis will be performed also for the per-protocol population. The intent-to-treat population will consist of all randomized subjects who signed informed consent. The per-protocol population will exclude all subjects in the intent-to-treat population who:

1. Have not undergone CEA
2. Have not received sonolysis during carotid CEA for at least 40 min
3. Have not attended one of the last 3 visits – visit 3 (24 h after CEA), visit 4 (30 days after CEA) or visit 5 (1 year after CEA)

Safety analyses will be performed on all randomized subjects undergoing the carotid intervention.

### Efficacy and safety endpoints

Primary efficacy endpoints:

1. Incidence of stroke or TIA during 30 days after the CEA in the sonolysis and control groups
2. For the MRI substudy: the incidence of new ischemic lesions on brain DWI-MRI performed 24 h after the CEA in the sonolysis and control groups

Secondary efficacy endpoints:

1. Occurrence of death, any stroke, or myocardial infarction within 30 days (myocardial infarction is defined as a post-interventional cardiac troponin T level increase of more than twice the normal upper limit in addition to either chest pain, symptoms consistent with cardiac ischemia, or electrocardiographic evidence of ischemia)
2. Changes in cognitive functions as evidenced by ACE-R, MMSE, Clock-drawing Test or Verbal Fluency Test scores 1 year post procedure relative to pretreatment scores
3. Changes in cognitive function as evidenced by ACE-R, MMSE, Clock-drawing Test or Verbal Fluency Test scores 24 h and 30 days post procedure relative to pretreatment scores
4. For the MRI substudy: number of new lesions and occurrence of new lesions ≥0.5 mL on post-procedural brain DWI-MRI
5. For the MRI substudy: incidence of ipsilateral new ischemic lesions on post-procedural brain DWI-MRI

Safety endpoints:

1. Safety will be evaluated with a summary of adverse events
2. Incidence of intracranial bleeding (including brain microbleeds) on T2 star-weighted GRE-MRI

### Statistical methods

The normality of distribution of all proceeded data will be checked using the Shapiro-Wilk test. Data with a normal distribution will be reported as the mean ± standard deviation. Parameters not fitting a normal distribution will be presented as the mean, median and interquartile range. Categorical variables in the two arms will be compared by Fisherʼs exact test. Continuous variables will be compared by the Studentʼs *t* test for normally distributed values, or the Mann-Whitney *U* test. Spearman correlation coefficient and intraclass correlation coefficient will be calculated for the evaluation of interobserver and intraobserver agreements of brain infarction volume measurement. Multiple logistic regression analyses will be used to determine the possible predictors of stroke or TIA, cognitive decline, or a new brain infarction. All tests will be carried out at a 0.05 alpha level of significance. All statistical tests will be performed at the Department of Biophysics, Informatics and Biometry, Faculty of Medicine and Dentistry, Palacký University, Olomouc.

### Collection of data to study database

All data will be collected by investigators during patients’ visits from the patient database and from the hospital electronic database. Only the investigators who will perform the randomization and/or sonolysis/sham procedure will add all patient data to the electronic REDCap database (https://kcentsrv.fnmotol.cz/redcap/redcap_v6.9.3/index.php?pid=16). This database will be used to generate tables and results to be executed by a statistician and will contain only encrypted information about the allocation group for each patient to maintain allocation concealment.

### Allocation concealment

Randomization and allocation to sonolysis or the sham procedure will be performed by the sonographer performing sonolysis or the sham procedure after the baseline visit. All randomized patients, neurologists performing neurological examinations at the baseline visit (visit 1) and control visits (visits 2, 3, 4 and 5), investigators performing cognitive tests at subsequent visits (visits 2, 3, 4 and 5), surgeons performing CEA and radiologists performing brain MRI will be blinded to allocation (sonolysis or the sham procedure).

### Adverse events

Adverse events (AEs) will be recorded for enrolled participants at any visit, including unscheduled visits. AEs are classified according to their relationship to the device and/or procedure and according to severity. All AEs, regardless of the reason or severity, are followed with appropriate corrective actions by the investigators until a satisfactory resolution is obtained. All AEs will be reported to the local Ethics Committee (Institutional Review Board) and the State Institute for Drug Control.

### Dissemination plan

The trial protocol was written following the Recommendations for Interventional trials (SPIRIT) Checklist (see Additional file 1). Schedule of enrolment, interventions and assessments are displayed in the Figure 1. The study findings will be reported according to the Consolidated Standards of Reporting Trials (CONSORT) guidelines (see Additional file 2). Protocol modifications will be reported when disseminating findings. Authorship of scientific articles emerging from the study will be decided upon by the International Committee of Medical Journal Editors’ guidelines.

## Discussion

The indications and safety of CEA and carotid stenting are still important and serious current topics, especially indications and safety of both methods. Thus, more studies are needed in this field. The optimal treatment procedure and medical management to avoid brain lesions during carotid revascularization is still under research [30].

Three recent meta-analyses concluded that sonolysis was a promising treatment for patients who have suffered from acute ischemic stroke [20, 31, 32]. Furthermore, the SONOBUSTER trial has demonstrated the effect of intraoperative sonolysis on the reduction of the prevalence and volume of new brain ischemic lesions after CEA and carotid stenting [22]. Results of the SONORESCUE trial have also demonstrated a significant reduction in the prevalence of larger new ischemic lesions and lesion volume in the brain after cardiac surgery [29]. The possible usability of sonolysis in the prevention of brain lesions during carotid revascularization was highlighted in the recently published review paper “The year in cardiology 2015: peripheral circulation” [30].

The mechanisms of sonolysis in the present study include acceleration of enzymatic fibrinolysis by the direct activation of a fibrinolytic system and increased transport of fibrinolytic agents (e.g., plasmin) into the thrombus by mechanical disruption of thrombus structure, destruction of gaseous bubbles, and transient peripheral vasodilatation [12–22, 29, 31, 32].

The study results will be important not only for the possible reduction of clinical vascular events, silent brain infarction or cognitive decline after CEA, but should also improve the understanding of the sonolysis mechanisms that accelerate fibrinolysis.

### Study limitations

Serial follow-up FLAIR and DWI-MRI will not be conducted to compare the progression or persistence of ischemic lesions. The cognitive test battery is limited to ACE-R. A more expanded neuropsychological battery would be more sensitive for the detection of specific cognitive impairment, but would be more time-consuming. This might also increase the risk of participants refusing to continue in the study or to complete the full battery, especially shortly after the surgery.

### Trial status

At the time of manuscript submission, 137 participants in 13 European centers have been recruited to the trial and it remains open to recruitment. The study was approved by the multicenter Ethics Committee of Vítkovice Hospital and local Ethics Committees of all participating centers (Ethics Committee of the University Hospital Ostrava, Ethics Committee of the Military University Hospital Prague, Ethics Committee of the University Hospital Motol, Ethics Committee of the Na Homolce Hospital, Ethics Committee of the Faculty Hospital Nitra, Ethics Committee of the University Hospital Martin, Ethics Committee of the Liberec Hospital, Ethics Committee of the České Budějovice Hospital, Ethics Committee of the T. Baťa Hospital, Ethics Committee of the University Hospital Plzeň, Ethics Committee of the University Hospital Hradec Králové and Ethics Committee of the Jihlava Hospital).

## Additional files

Additional file 1: SPIRIT Checklists with recommended items to address in a clinical trial protocol and related documents. (DOC 122 kb)
Additional file 2: CONSORT 2010 Checklist of information to include when reporting a randomized trial. (DOC 218 kb)

## Abbreviations

ACAS: The Asymptomatic Carotid Atherosclerosis Study
ACE-R: Addenbrooke’s Cognitive Examination Revised
CEA: Carotid endarterectomy
CTA: Computed tomography angiography
DSA: Digital subtraction angiography
DWI: Diffusion-weighted imaging
ECST: The European Carotid Surgery Trial
EPI: Echo planar imaging
ETL: Echo train length
FLAIR: Fluid-attenuated inversion recovery
FOV: Field of view
GRE: Gradient-recalled echo
ICA: Internal carotid artery
MCA: Middle cerebral artery
MMSE: Mini Mental State Examination
MRA: Magnetic resonance angiography
MRI: Magnetic resonance imaging
mRS: Modified Rankin score
NASCET: The North American Symptomatic Carotid Endarterectomy Trial
NIHSS: National Institutes of Health Stroke Scale
SONOBUSTER: The Sonolysis in Prevention of Brain Infarctions during Carotid Stenting and Carotid Endarterectomy trial
SONORESCUE: The Sonolysis in Prevention of Brain Infarction during Cardiac Surgery trial
TCD: Transcranial Doppler monitoring
TIA: Transient ischemic attack
TIC: Thermal Index for cranial bone
